# More Things in Heaven and Earth: Spirit Possession, Mental Disorder, and Intentionality

**DOI:** 10.1007/s10912-018-9519-z

**Published:** 2018-07-19

**Authors:** Mohammed Abouelleil Rashed

**Affiliations:** 1 Department of Philosophy, Birkbeck College, University of London, London, UK; 2 Department of Philosophy, King’s College London, London, UK

**Keywords:** Intentional stance, Spirit stance, Madness, Daniel Dennett, Derek Bolton, Jinn

## Abstract

Spirit possession is a common phenomenon around the world in which a non-corporeal agent is involved with a human host. This manifests in a range of maladies or in displacement of the host's agency and identity. Prompted by engagement with the phenomenon in Egypt, this paper draws connections between spirit possession and the concepts of personhood and intentionality. It employs these concepts to articulate spirit possession, while also developing the intentional stance as formulated by Daniel Dennett. It argues for an understanding of spirit possession as the *spirit stance*: an intentional strategy that aims at predicting and explaining behaviour by ascribing to an agent (the spirit) beliefs and desires but is only deployed once the mental states and activity of the subject (the person) fail specific normative distinctions. Applied to behaviours that are generally taken to signal mental disorder, the spirit stance preserves a peculiar form of intentionality where behaviour would otherwise be explained as a consequence of a malfunctioning physical mechanism. Centuries before the modern disciplines of psychoanalysis and phenomenological-psychopathology endeavoured to restore meaning to 'madness,' the social institution of spirit possession had been preserving the intentionality of socially deviant behaviour.

## Introduction

Spirit possession refers to a broad range of phenomena whose basic defining feature is the involvement of a non-corporeal agent with a human host in a variety of ways. These agents— commonly referred to as spirits—may be ghosts of departed ancestors or foreign visitors, divine beings, demons, spirits of fire; in general, ethereal creatures of various origins.^1^ Spirit possession is ubiquitous in almost all regions of the world. In a cross-cultural survey published in the 1960s, anthropologist Erika Bourguignon (1968) documented the presence of institutionalised possession in 74% of the societies included (360 out of 488 societies). In Sub-Saharan Africa and the Circum-Mediterranean (which includes North Africa) the figures were higher than the average, 81% and 77% respectively.^2^ Judging by more recent ethnographies, reports and reviews, and my own research in Africa, the prevalence and everydayness of spirit possession in many communities are not waning (e.g. Boddy 1994; Cohen 2007; Rashed 2012). In these societies, spirit possession is not only an explanatory theory for illness; it informs people's understanding of themselves and others in such domains as agency, responsibility, identity, normality, and morality.

In this paper I draw some connections between spirit possession and the concepts of personhood and intentionality, prompted by my engagement with the institution of spirit possession in Egypt. Considerations of spirit possession offer an occasion to articulate a perspective on the phenomena that makes use of the aforementioned concepts, while at the same time extending understanding of the variety of intentional explanation/prediction of behaviour as the latter had been formulated by the philosopher Daniel Dennett.^3^ Specifically, I argue that spirit possession—or as I shall call it the *spirit* stance—is a variant of the intentional stance in that it aims at predicting and explaining behaviour by attributing to an agent (the spirit) beliefs and desires but is only deployed once the behaviour of the subject (the person) is judged to have failed specific normative distinctions. Applied to behaviours commonly associated with mental disorder, and in contrast to an every-day disenchanted folk psychology, the spirit stance preserves some intentionality where the alternative is likely to be an explanation of behaviour as a consequence of a dysfunctional physical or psychological mechanism.

I proceed by exploring ways of approaching spirit possession, cognizant of the affinities between *possession* and dissociative phenomena, and the apparent metaphysical impossibility of s*pirit* possession. After treading a cautious line through these issues, I attend next to personhood. I begin with a vignette describing a case of spirit possession and continue by arguing that the attribution of beliefs and desires to 'spirits' arises from their representation as persons. By appealing to contemporary debates on personhood, I demonstrate that in the manner they are represented, spirits possess many of the requirements considered essential to personhood. I then outline the different ways in which knowledge pertaining to a specific spirit-person is gained, for instance the spirit's name, gender, traits, and dispositions. Having articulated the status of spirits as persons, I proceed to describe the connections between spirit possession and intentionality. I present a brief outline of Dennett's conception of intentional systems, the development of this theory by Derek Bolton, and its application to mental disorder. With the ground prepared I present the proposal for the spirit stance. The remainder of the paper is then devoted to explaining and clarifying how the spirit stance works, and responding to some potential objections.

## How are we to approach spirit possession?

The involvement of spirits with their human hosts is understood by adherents and practitioners to take various forms. The spirit may intrude into the person causing physical and psychological maladies or, less commonly, generating positive effects such as heightened capacities and powers. Social misfortunes such as financial problems and interpersonal discord may also be attributed to spirit influence through the effects of the spirit on the person's mental states. The involvement of spirits with their human hosts is not limited to the effects of intrusion and may manifest in displacement of the host's agency. This displacement may be complete, in which case the spirit's identity and agency effectively replace that of the person, whose physical body now becomes a vehicle through which the spirit(s) speaks and acts. Or it may be partial, in which case only certain actions are understood to emanate from the spirit's agency. With full displacement, the person—typically, but not always—would not have conscious awareness for the duration of the episode, a state commonly referred to in the literature as a trance state. Following Cohen's (2008) typology, I will refer to intrusion (whatever the effects) as *pathogenic* possession and to displacement (whether partial or total) as *executive* possession.^4^ Executive possession is particularly important for the institution of spirit possession as it is a central means by which the identity of the spirit can be known through conversing with it. Given this brief outline, how do we approach spirit possession?

Pathogenic possession may be the easier of the two to approximate as it resembles what we would normally think of as a causal attribution theory of illness. For example, instead of explaining a depressed mood by citing a neuro-chemical imbalance, the person would do so by citing the effects of a spirit. The explanation may stop there without any specification of a detailed causal pathway. But I found in my research that healers sometimes employ a representation of human biology of various degrees of sophistication to argue that spirits achieve their effects by directly targeting the bodily organ or centre responsible for that effect (Rashed 2012). In any case, pathogenic possession can be thought of as a theory of illness based on the idea of the intrusion of an agent (e.g. virus, carcinogen) into the body, albeit the causal agent here—the spirit—is one that many would object to on various grounds. I will address how we can approach the *spirit* component of both forms of possession towards the end of this section. But first, what about executive possession?

Executive possession is a familiar albeit fringe notion in modern popular culture. The idea that a person's agency and identity can be displaced or eclipsed by an incorporeal agent is the subject of many movies, features in the historical record, and is currently endorsed and practiced by certain churches in the form of demonic possession. Even though it is a familiar notion, it remains one that resists understanding by its apparent exotic nature. How are we to approximate possession within a naturalistic view of the world? Possession, at the very least, makes a statement pertaining to agency. As Vincent Crapanzano (1977) had expressed, possession serves as

a very powerful metaphor for the articulation of that range of experience in which the subject feels “beside himself,” not fully responsible for his own condition, as in extreme love, intense hatred, tantrums, furore, excessive courage, compulsive ideation, the *idée fixe*, obsessional acting out, and, of course, fascination itself. (7)

Metaphorical as that may be, the idea is that when one is intensely in love or obsessional about an object, one is moved by emotions and compulsions powerful enough to evoke the experience of being driven if not against one's will then against one's rational judgment. However, executive possession has a further component of identity switch, which implies a partial or total loss of agency vis-a-vis the identity in question—similar to Multiple Personality Disorder (MPD) or, as it is now known, Dissociative Identity Disorder (DID). In DID the person has several alters, one of which dominates the others (or one’s core identity) at any given moment. While the imaginative leap from possession-as-infatuation to DID may seem too great, the seeds for conceiving DID can already be found in possession-as-infatuation. To be driven against one's rational judgement is a few steps removed from being driven against one's conscious will. The latter is an experience of a source of agency within us that is sufficiently distinct so as to become salient. Through various imaginative increments of objectification and alienation we can see how that source of agency may be identified with a persona. This persona may acquire independence with dispositions of its own, responsible for certain actions and emotions: it becomes an alter. Perhaps we can conceive a continuum of possession states from the more familiar pull of infatuation to the unnerving cases of DID. The continuum does not suggest a shared causal structure to these phenomena, only that they can be seen as gradations of each other.^5^

Depth psychology accounts for the full range of possession phenomena without having to posit any outlandish beings. Depth psychology refers to any theory that posits a layered psyche with hidden motivations and processes and which is capable of deceiving itself or, in extreme situations, of fragmenting. For instance, a typical explanation for DID would cite the impact of childhood abuse on ego development such that splitting (dissociation) becomes the primary response to severe distress. Conversely, a typical explanation for DID by a Qur'anic healer in the Western desert of Egypt is, in some ways, simpler: the person has been possessed by a spirit that had targeted him or her due to sorcery, attraction, bad luck or some such reason. There is no splitting in this case, distress need not be a precipitating feature, nor are childhood experiences necessarily relevant. For the psychologist the 'entity' is part of the ego (where else would it come from?), while for the Qur'anic healer it is external to the subject. This is reflected in treatment strategies: psychological treatment usually consists in managing the different personalities by fostering awareness and communication among them, seeking their integration, or cultivating the original 'core self' (see Littlewood 2004). While in spirit possession interventions range from exorcising the spirit to developing an ongoing relationship with it by which the host may become a medium.

The similarities between DID and spirit possession have long been noted: both evince radical identity alteration and discontinuity, total or partial loss of control over behaviour, and limited memory of such states (Bourguignon 1989). Writing from a historical perspective, Kenny (1981, 1986) observes that in 19^th^ century spiritism, interpretations of what we would now call DID included the idea that individuals were possessed by spirits. The decline in belief in spirit possession has seen a concurrent decline in such phenomena. The return of DID to Europe and America in the second half of the 20th century was in the context of a developed depth psychology that could no longer see DID as the incarnation of external agents but as the manifestation of an ego forced into such contortions by childhood abuse. This perspective gained popularity through publicised cases, books and movies, bringing with it the problem of false memories of abuse (Littlewood 2004). The idea of possession by demonic and alien entities can still be found today among some British and American psychiatrists, doctors and clergy (ibid.). On the basis of descriptive and phenomenological similarities we can consider MPD/DID and spirit possession to be, at least in these respects, equivalent phenomena.

Having partially approximated the notion of pathogenic and executive possession within a naturalistic worldview, there remains an important question: what about the spirits? Is spirit possession a dissociative identity disorder in which the alters are conceived as super-natural? Is spirit possession a phenomena in its own right mediated by other-worldly entities? Can spirits be blamed for the illnesses and maladies they supposedly cause? The answer to these questions will depend on many things but mainly on our metaphysical commitments; they amount to asking if spirits and *spirit* possession are possible. A materialist ontology, naturally, would deny this possibility. In fact this is the assumption implied by almost every single scholarly work on spirit possession.^6^ Something like: given that spirits do not exist, how then do we explain/understand what is going on when people say they are possessed by spirits? The psychological theory of dissociation is, at present, a popular answer for executive possession. And for pathogenic possession there are numerous theories at our disposal to explain the effects in question. But, really, what about the spirits?

Consider the physicalist doctrine that any state that has physical effects must itself be physical. This doctrine leaves two options for those who wish to defend spirits, neither of which is promising. On one hand if they insist that spirits do have effects in the physical world they would have to concede that spirits are not, after all, the ethereal creatures they are claimed to be: they are either physical or supervene on the physical. On the other hand if they concede that spirits do not have effects in the physical world (and hence *spirit* possession is not possible) while maintaining that they exist outside the causal realm, the very possibility of spirits becomes questionable on epistemological grounds. The problem here is that an entity that cannot have any physical effects poses epistemological problems: how else would we know about it if not through our senses, which requires of such entities to be capable of influencing the physical world?^7^ In fact, *spirit* possession is probably only possible given a substance-dualist interactionist ontology: Cartesian Dualism. Spirit possession requires that there are two distinct substances in the universe (material/physical and immaterial/spiritual), and that two-way causal interactions between these substances are possible. Displacement of the human host's mind/soul by the spirit would then be a switch of immaterial substances which assume control of the physical body. However, interactionist dualism is not a popular view in philosophy despite being an everyday, common-sensical view: the physical world affects our thinking and emotions, both of which affect our actions.^8^ It also remains essential to monotheistic theology.

If we are tempted by physicalism, then it is unlikely that spirit possession is possible. On the other hand, if we are committed Cartesians, then we might have other objections to spirit possession—say the nature of *spirits*—but it won't be its *prima facie* impossibility. We may assume that physicalism is true, in which case what is called *spirit possession* is just a fancy DID (executive possession) or a mistaken theory of illness (pathogenic possession). This position, in my view, diminishes our inquiry into spirit possession. I propose that despite descriptive and phenomenological similarities between spirit possession and DID, and despite the fact that scientific explanations of illness are often superior (prediction, outcome), we have reason in many instances not to reduce spirit possession to either. This claim does not arise out of respect for alternative worldviews—important as that may be—nor is it out of aesthetic preference for a term over another: spirit possession embodies moral, social, practical, and psychological consequences entirely different to the reductive nature of the disenchanted *psy* disciplines.^9^ For instance, in DID, the person is expected to grapple with persons/identities that, according to current psychological wisdom, his own mind had created. By contrast to this myopic focus on the person, spirit possession immediately places the possessed in a much wider interpretive, experiential, and social space: in a prior existing and developed institution. Boddy (1994) expresses this well in relation to biomedical, but I may also add psychological, frameworks:

Unlike biomedicine, which collapses into the body, possession widens out from the body and self into other domains of knowledge and experience—other lives, societies, historical moments, levels of cosmos, and religions—catching these up and embodying them ... Phenomena we bundle loosely as possession are part of daily experience, not just dramatic ritual. They have to do with one's relationship to the world, with selfhood - personal, ethnic, political, and moral identity. (414)

In what follows I offer a perspective on spirit possession that makes use of the philosophical concepts of personhood and intentionality. I shall extend understanding of the variety of intentional explanation and prediction of behaviour, and of the kind of work spirit possession can do in a community. The aim is partly to reveal what can be learnt from the remarkably resilient and widespread institution of spirit possession, especially with regards to behaviours that are taken by societies around the world to imply 'madness' or 'mental disorder.' I assume for the sake of exposition that there are spirits and that spirit possession is possible, and resist reducing either to psychological or biological categorization. Eventually I bring things back to earth by examining the implications of this exercise for a range of concerns. For now, however, I urge the reader to suspend disbelief and to accept that there are more things in heaven and earth than are dreamt of in our philosophy. I begin with a short story.

## Spirits and personhood

*Girgis is a fifty-year-old Coptic-Christian male who lives with his wife and two children at the far end of the oasis where you can see the edge of the desert. He became involved with a farmer who had unknowingly trespassed upon and damaged his habitat. Angered by this incident and by the damage sustained to his home, Girgis began harassing the farmer. He would wake him up at night, put him in a bad mood all day, prevent him from praying at the mosque, and generally make everything difficult for him. The farmer sought one of the local healers to intervene and arbitrate between them. The healer agreed to do so, and upon meeting with Girgis, he reminded him that both Christians and Muslims are people of the Book and should not harass each other like this. He assured Girgis that the farmer had no intention of trespassing upon his habitat, and that it is time to end this misunderstanding.*^10^

The reader may be surprised to learn that Girgis is not a human person; he is a spirit of a variety known in Egypt and in Muslim societies across the world as a *jinni* (plural *jinn*). Despite not being *human* persons, spirits are represented as persons. They are deemed to display features required for personhood, and it is on the basis of these features that people in the community consider it possible to reason with them.

Providing a set of necessary and sufficient conditions for personhood is fraught with difficulty and disagreement, and it would seem that there are several, as opposed to one, concept of the person (see Braude 1995, ch.8). Features that are commonly put forward include the following: a person is a member of a "significant and ordered collectivity" (Carrithers 1985) pertaining to which the entity in question has (or will have) rights and towards which it has (or will have) obligations. It is considered a requirement for this sense of personhood that the entity must be capable (now or at a future time) of practical reasoning: of generating goal-directed action through deliberative reflection. Moreover, some accounts require that a person must not only be capable of acting on the basis of reasons, but must have a sense of oneself as an agent for whom things matter in accordance with certain standards. Taylor (1985) calls these standards the "particularly human significances" such as shame and guilt (263).

Requirements for this sense of personhood are not met by all individuals, for example those with severe brain damage or who are in a coma. Braude (1995) distinguishes this sense of personhood from what he refers to as the *forensic* concept of the person (194). This refers to entities that do not have the capacity for practical reasoning—and who thus might be free of obligations—but who nevertheless are, or should be, considered bearers of rights. Current debates on the moral status of individuals with severe cognitive impairment and certain non-human animals can be understood as pertaining to the forensic concept of the person (see Kittay and Carlson 2010). These debates have become an occasion to revise what we take to be constitutive of (forensic) personhood. A recent account, for example, argues that the capacity to care rather than the capacity for practical reasoning should be the basis for ascribing to others moral status as persons (Jaworska 2010). Recognition of forensic personhood evinces cultural and historical variation. In some societies, attributions of personhood admit of a temporal process and are part of an ongoing “moral career” culminating in a series of initiation rites (Harris 1978, see also 1989). Historically, personhood was denied certain individuals on the basis of their status as slaves (Mauss 1985). In both cases, the individual may be capable of practical reasoning but is only recognised as a person, and hence worthy of respect, on completion of the relevant initiation rites or after being granted his or her freedom.

The assumption in the previous discussion has been of a one to one correspondence between a person and a living organism (see Braude 1995, 199). However, certain conceptions of the person do not require this. Of note is the fact that in many cultures and religious traditions entities considered persons can inhabit many bodies and one body can be inhabited by several persons. Moreover, personhood and embodiment come apart. Spirits, as indicated earlier, are regarded as disembodied persons who are able to acquire executive control of a human individual. But acquiring a body does not add to their status as persons. This status is evident if we consider the manner they are represented and which fulfills several of the criteria listed above. The *jinn* are members of a significant and ordered collectivity: they are socially organised, work, marry, and procreate. They are gendered, have human-like traits and concerns. They are capable of goal-directed action and possess moral agency which renders them subject to trial and punishment. It is by virtue of these features that it is possible for the healer to reason with them and to appeal to their sense of right and wrong as the vignette above demonstrates. The *jinn* also enjoy recognition as persons in the forensic sense. Thus, healers are wary of harming the spirits in so far as it is not necessary to do so, and this stems not only from fears of retaliation, for instance, but from an understanding that spirits are persons and are, at least, worthy of respect on that basis. By contrast to the *jinn*, in Islam, angels are not persons; they are emanations of god's will and hence are incapable of agentic behaviour.

Given their status as persons, how do people attain knowledge of these spirits? How is the general and impersonal category 'spirit' individualised into a specific *spirit-person* with an identity, name, gender, religion, history, traits, dispositions, and intentions?

## Gaining knowledge about spirits

Observations of spirit possession in Egypt demonstrate that knowledge about spirits is gained through various modalities each with its own claim to certainty and level of detail: religious texts, traditions and social interaction, direct communication, embodied experience, and frank emergence.

Religious texts such as the Qur'an and the compendiums of *hadiths* (sayings) attributed to the Prophet of Islam do speak of a category of being known as the *jinn.* The *jinn* are mentioned many times in the Qur'an, the most famous of which is a verse stating the purpose of their creation: "I have created jinn and mankind only to worship Me" (*Al-Dhariyat*: 56), and another usually interpreted as referring to harm accruing from "satan’s touch" (*Al-Baqara*: 275).^11^ Nevertheless, the extent of the attribution of illness and misfortune to spirits and the more colourful ways of exorcising them cannot be accounted for through the content of the Qur'an, though they do have a basis in some *hadiths*. For believers, such texts while they are high on certainty are nevertheless low on detail as they can only offer knowledge of a general nature. By contrast, the oral history of the community and the exchange of stories pertaining to recent or present experiences of possession, offer more detail about the nature of spirits and how they behave.

The remaining three modalities all involve an experience of the spirit rather than hearing a story about it from other sources. As the name implies, direct communication pertains to persons having auditory and visual experiences of certain spirits, thereby coming to learn about them. Embodied experience and frank emergence may occur spontaneously or at a healing session. Consider this typical description of a diagnostic and healing session as would be conducted by a Qur'anic healer in the community I studied. With his right hand placed over the subject's forehead, the healer reads loudly the *ruqya* (incantation of specific Qur'anic verses) and registers the subject's response: four possibilities are recognised. The first possible outcome is that the *jinni* emerges and animates the subject's body, whose voice and identity are now replaced. The healer proceeds, through conversing with the *jinni*, to identify his or her name, religion, whether or not there is sorcery, the reasons behind possessing the subject, intentions at the present moment, and other questions relevant to getting to know the spirit. The healer then proceeds to negotiate with the spirit and secure its exit from the human host. The second and most common outcome is that the person responds with symptoms and signs such as mild tremors or numbness in the limbs, headache, screaming, stiffness, blurring of vision, arousal, violence, attempts to leave the room, crying, or perhaps would be seen scanning the room in disdain and with an incongruent smile. Any of these are sufficient indications that a *jinni* is involved.^12^

Applying this to the vignette mentioned previously we find the following: initially the farmer experienced insomnia and dysphoria. He suspected spirit interference (pathogenic possession) and went to the healer who administered the incantation. A *jinni* emerged (executive possession), and the healer began conversing with it. This is how the healer was able to learn the *jinni's* name, religion, and understand the circumstances that occasioned the possession incident. Note that knowledge regarding the spirit's intentions can already be suspected from more general information pertaining to it. For example, a pagan *jinni*—in this community—is regarded as potentially dangerous as it would have no regard for God and religious morality; it would have no qualms to harm the host or to behave in capricious ways. On the other hand for a Muslim host, a Muslim *jinni* is generally considered less likely to harm the host or behave insolently, and is easier to negotiate with by appealing to his or her sense of right and wrong.

The exposition, so far, sought to portray spirits as social persons who may interact with humans under various circumstances. Their identity as beings with such and such traits and capable of agentic behaviour is demonstrated and further refined when a spirit displaces the host's agency and makes its presence explicit or otherwise directly communicates with the host. This is how spirits are perceived in some societies where the institution of spirit possession exists. In order to further understand spirit possession and appreciate some of its consequences in relation to behavioural disturbances, I will introduce for this purpose Daniel Dennett's conception of the intentional stance, and the development and application of his theory by Derek Bolton in the case of mental disorder.

## Mental disorder and attributions of intentionality

According to Daniel Dennett (1981, 1987), we can assume three stances to explain or predict the behaviour of an organism or machine—a system. From the physical stance we appeal to our knowledge of the physical constitution of the system and the laws that govern its conduct. From the design stance we assume that the system has a particular design and that it will function as designed; we do not require, for this purpose, knowledge of the physical implementation of the functions in question. From the intentional stance we attribute to the system beliefs and desires, and by assuming that it is rational—i.e. optimally designed relative to goals—we predict that it will act to further its goals in light of its beliefs and desires. The intentional stance underpins the power of folk psychology at providing predictions of other people's behaviour as well as of some higher animals and complex machines such as chess-playing computers. It is the stance most commonly adopted in everyday interaction with others. Dennett (1987) notes that there will be cases beyond the power of the intentional stance to describe and, by way of analogy, cites the difficulty in discerning the behaviour of an artefact from its design if the artefact is physically damaged (28). In the case of human beings he implies that fatigue and malfunction may similarly hamper prediction from the intentional stance (ibid.). When there is such breakdown in function, Dennett (1981, 5) suggests, we drop to the physical stance to explain behaviour.

This idea has been substantially remarked upon and developed by Derek Bolton (Bolton 2001; Bolton and Hill 2004) in the context of the apparent absence of intentionality that is generally considered a hallmark of mental disorder. Starting with the point that failure to recognise intentionality in the mental states and actions of others underpins attributions of 'madness,' he points out that attributions of intentionality are observer-relative (Bolton 2001, 187). Upon encountering activity, different observers "may see different patterns of intentionality at work, including the vacuous case of seeing no such patterns" (Bolton and Hill 2004, 98). The assumption that apparent lack of intentionality signals physical dysfunction may thus indicate hastiness in dropping to a lower level explanation (2001,188). Bolton then proceeds to demonstrate that there are a number of options from within the design as well as the intentional stances to explain breakdown in function. That aside, the key point here is that the intentional stance is abandoned when the mental states or activity in question fail certain normative distinctions *as judged by the observer*. Bolton and Banner (2012) express some of these distinctions as applied to action and various mental faculties:

Perception of reality can be veridical or mistaken, or in an extreme, hallucinatory. Beliefs may be true or false, reasonable or unreasonable, based on good evidence or otherwise. Desires are reasonable or otherwise depending on their relation to the person’s needs. Emotions may be understandable reactions to events, for example, anger is an understandable response to being hurt, or not understandable, being angry for no reason; and so on. The will may fail to control action. Action may be reasonable or otherwise, depending on whether it follows from beliefs and desires, or on whether those beliefs and desires are themselves reasonable. Behaviour may be random, without any relation to the achievement of goals, without method, and in this sense may fail to be real action... (83)

The observer relativity and hence the wide range of possible evaluations and interpretations at each of these faculties is evident. Different observers may see in a child's tantrum an attempt to coerce the parents to provide yet another toy or in that same behaviour merely that the child is 'tired.' In the first case intentionality is still at play, in the second the parents are (perhaps wisely) reluctant to pursue it. Observer relativity also has a cultural dimension. An example, further discussed below, is the tendency in some societies to see certain emotions— say unhappiness in a marriage—as having nothing to do with the personalities involved or other relational issues, but rather as states imposed by an interfering spirit. Many readers are likely to understand interpersonal emotions as having to do with the person and the relationship.

The idea I want to pursue in what follows is that spirit possession—or as I shall call it henceforth the *spirit stance*—occupies a peculiar position: it is an intentional strategy in the sense that it aims at predicting and explaining behaviour by ascribing to an agent (the spirit) beliefs and desires, but it is only deployed once the mental states and activity of the subject (the person) are deemed to have failed normative distinctions of the sort just outlined. It thus subverts the person's agency, while simultaneously maintaining a peculiar form of intentionality where otherwise one might expect a drop to the physical stance. Whether it achieves this and the manner by which it does will be subsequently discussed. First I will describe some of the situations in which the spirit stance is adopted and the normative distinctions that occasion this. These examples will serve to illuminate the way in which the spirit stance cuts across the ascriptions of what may be described as a disenchanted folk psychology.

## Encounters with spirits in Egypt

For both the healer and the possessed person, a question arises as to why the spirit had targeted that person in particular. In the Dakhla oasis of Egypt, where I had conducted research, three answers are available. The first is bad luck, such as in the case of the farmer (cited above) who inadvertently stepped upon a *jinni's* habitat. The second is infatuation (*'eshq/mekhaweyya*): a spirit is attracted to and selects a human host. The third, and most common, is sorcery (*se'hr*): here a person who would like to see another disadvantaged visits a sorcerer who is able to direct a *jinni* at the victim. The *jinni* is instructed to wreak havoc usually in a specified domain—physical health, behavioural, psychological—with the final purpose of imposing various sorts of social failures (e.g. problems at work, marital discord, impeded marriage possibilities, impotence). Whatever the means by which person and spirit are brought in proximity, the understanding is that a person is made more vulnerable to possession if he or she fails to secure protection through prayer and other forms of worship.

The spirit stance is adopted to explain a wide range of behaviours and is certainly not limited to 'illness.' Table 1 illustrates some examples from Dakhla, together with an indication of the normative distinctions that the behaviours or mental states are deemed to have failed. In each of these cases, un-understandability, unreasonableness, inappropriateness, etc., signal that the mental state or behaviour in question is imposed from without, hence deployment of the spirit stance.

**Table 1:** Socially deviant predicaments and accompanying normative distinctions in the community

	Description	Normative Distinction
*Marital discord*	Quarrels and dysphoria in the context of marital discord may be attributed to the effects of a *jinni* directed at the husband, the wife, or both through sorcery.	Psychological states and behaviours are deemed unreasonable or un-understandable given the nature of the relationship; there is no reason for such discord.
*Unorthodox love*	A man’s excessive desire for and obedience towards his spouse is attributed, almost always by his brethren, to the effects of a *jinni* directed through sorcery in what amounts to a plot arranged by the woman, or someone else, to control him.	Emotion and desire are deemed inappropriate; it is not appropriate to desire a woman (or this woman specifically) to this extent.
*Inability to marry*	Applies to men and women who move into their thirties without getting married. Dysphoria in the presence of suitors, or frequent rejection of eligible ones, can be understood to arise from the influence of a *jinni* usually directed through sorcery.	Psychological states and behaviours are deemed unreasonable given the situation; there is no reason for him or her to reject this appropriate, potential spouse.
*Unwanted compulsions*	Socially unsanctioned desires such as homosexual urges are attributed by their bearer to a *jinni* directed through sorcery.	Compulsions and desires are judged inappropriate; one cannot desire a member of the same sex.
*Madness*	Aimless roaming, frequenting deserted places, preoccupation with fire, talking to oneself, lack of attention to appearance, inability to remain in one place for long, isolation, aggression, and sleeping outdoors are some behaviours that attract a spirit stance explanation of 'madness'.	Behaviours are deemed inappropriate and/or un-understandable; no normal person would behave like this.

In order to draw out the implications of the spirit stance it is helpful to have a view on what to contrast it with. I will take the contrast to lie in a disenchanted folk psychology, the kind, for example, where interpersonal conflict is usually explained by consideration of the personalities involved and, say, their temperaments. It is also one in which 'madness' tends to be seen as a consequence of dysfunctional physical or psychological mechanisms. Given this, and in light of the preceding examples, it can be seen that the spirit stance cuts across ascriptions of such a folk-psychology: it extends into areas that would normally—though by no means exclusively—be described from the intentional stance (marital discord, other social and interpersonal problems), as well as into areas that would normally be described from the physical stance ('madness,' mental disorder). We can say that in both areas the spirit stance subverts the agency of the person but in the latter (mental disorder) it preserves another form of intentionality, where otherwise there may have been a drop to a physical stance explanation of the person's behaviour and mental states.

## The spirit stance in the explanation and prediction of behavior

The spirit stance is a variant of the intentional stance in that it explains the inappropriate or un-understandable behaviour of a human-agent by positing a non-corporeal entity now seen as the agent of this behaviour. To demonstrate how it works, consider behaviours that may attract a social judgement of 'madness'; a few have been listed in table 1. These will vary from one socio-cultural context to another. Now recall that spirits are (represented as) agents with beliefs, desires, and dispositions, capable of setting goals and acting on them. What does it mean to say that the person is behaving in this way because he or she is possessed by a spirit? The first sense in which this can be understood is executive possession; that is, the behaviour witnessed is literally the spirit's. As indicated at the beginning of this paper, displacement of the host's agency need not be accompanied by a trance state—an altered state of consciousness. Thus the behaviour is understood as intentional by virtue of the spirit's agency. Most generally, it would be said that it is in the nature of a spirit to seek deserted places and isolation, to be pre-occupied with fire, to be restless. The second sense in which behaviour is ascribed to a spirit is pathogenic possession. Here, the spirit is 'making' the person behave in those bizarre ways. While behaviour in this case is not, strictly speaking, the spirit's, it remains describable in an intentional idiom in those cases where sorcery is involved. Sorcery is a common reason why spirits become involved with human hosts. As practiced in Dakhla, sorcery typically involves three agents: the seeker (the person who wants the harm arranged); the sorcerer; and the spirit that will do the work. The purpose is to make the person behave in a 'mad' manner and thus to harm that person socially. The victim's behaviour is therefore goal-directed but the beliefs and desires that direct the behaviour, and the goals that are being served, have been established elsewhere in the nexus of relations that constitute sorcery.

In terms of prediction of behaviour, this requires that the applied theory (e.g. folk psychology) tracks some pattern in the world in order for predictions to obtain in actuality. What pattern does the spirit stance aim to track? Here we return to issues raised earlier when discussing approaches to spirit possession. If there are such things as spirits, then the spirit stance tracks the intentionality of spirit-persons in the same way that the intentional stance tracks the intentionality of human persons: assumptions are made concerning the beliefs and desires the agent ought to have and, being rational, that the agent will act to further its goals. Here, procurement of individualised knowledge pertaining to the dispositions and intentions of spirit-persons (as outlined earlier) will facilitate the prediction of behaviour. On the other hand if spirits do not exist, and the only source of agency is the person, then it is not clear how individualised knowledge of the spirit—now seen merely as a fiction of the person's mind arising during a trance episode or direct communication—can play any role in the prediction of behaviour. It would not matter what the 'spirit's' dispositions are as there is only one actor here: the person.

The only situation in which it may be possible to predict the person's behaviour by tracking the 'spirit's' intentionality is when the person actually takes on the dispositions and features of the spirit (or the unconscious/unacknowledged/alienated—however you would like to put it—part of his personality) he had come to learn about. And this actually does occur; consider these examples from the Dakhla oasis: a Muslim man possessed by a Christian spirit stops attending the mosque, begins reading the Bible and praying to Jesus; a woman possessed by a capricious, mischievous spirit behaves in such a manner where this is out of character for her. Pressel (1977) makes a similar point, here pertaining to the Brazilian Umbanda religion: "After learning to play the role of each spirit, the novice may extend that personality trait into his own everyday behaviour" (346). She cites the case of an "extremely impatient woman" she knew who "had learned to be calm from her *preta velha* spirit [spirits of old African slaves known for being peaceful, compassionate, patient, and wise]" (ibid). Thus, even if we reject spirits as possible agentic entities, there is still room for the spirit stance to allow for the prediction of a person's behaviour. This will depend on the extent to which the personality of the individualised 'spirit' is integrated by the person who supposedly is possessed by that spirit.

## Objections and clarifications

The proposal for a spirit stance raises some objections and requires further clarification. One objection concerns its presumed uniqueness. It could be argued that the spirit stance is really nothing but the intentional stance, only that the agent is distinguished from the person whose behaviour is being described. Alternatively, it could be pointed out that the spirit stance is really a physical stance as in many cases the person's behaviour is described through non-intentional processes (spirits enter the person and affect bodily organs). My argument in this paper has been that the spirit stance is a *variant* of the intentional stance. Hence, in response to the first objection, I agree that it is an intentional stance but not that it is thereby indistinguishable from it. The crucial point here is that the spirit stance is adopted only once the intentional stance is abandoned. The spirit stance includes the recognition that mental states and behaviour have failed certain normative distinctions—the reason the intentional stance is abandoned—yet continues to describe both in an intentional idiom. In response to the second objection—that the spirit stance is a physical stance—I agree that intrusion by spirits sounds a lot like, say, infection by viruses. And the latter is a common physical stance account for tiredness, moodiness, etc. However, as I have endeavoured to elucidate throughout this paper, spirits are represented as persons whose nature can be known and who are capable of intentional behaviour. That is why it is possible in some cases to explain as well as predict behaviour by positing such entities, irrespective of whether spirits are independent agents or cultural-psychological fictions.

Earlier I noted that the spirit stance subverts the person's agency by abandoning the intentional stance, yet preserves another form of intentionality mediated by the spirit-person. This thesis requires further remark. The subversion of agency need not be a conscious decision on behalf of the observer though it certainly can be; there is a thin-line separating the inability from the unwillingness to see intentionality in the behaviour of others. The examples listed in table 1—in particular those applying to relationships—might seem to a modern sensibility as blatant attempts to subvert action and mental states of their (inter)personal meanings in favour of an externally imposed efficient cause. For example, blaming marital discord on spirit influence and sorcery subverts the couple's moods of the usual interpersonal referents such as personality 'clashes'; the problem is not *in* the relationship. Now, some may find it problematic that a society disapproves of adulation towards one's wife and homosexual urges—the other examples in table 1—to the extent that they can only be understood as externally imposed states. However, in principle what is going on here is no different from what occurs in communities where there are no spirits: earlier I used the example of a toddler whose parents are unable to/do not wish to see in his tantrum anything more than tiredness. And we are all aware of pejorative references to 'hormones' when someone wishes to cast doubt on the rationality and intentionality of another's behaviour. The difference is not in kind, rather, it is in the values and the behaviours that attract non-intentional explanation. Abandonment of the intentional stance is common in everyday life, even if the reasons and normative distinctions that occasion this vary relative to cultural contexts and observers.

Turning to the second part of the thesis: that the spirit stance preserves a form of intentionality where otherwise one might expect a drop to the physical stance. This applies to inappropriate and un-understandable behaviour, as is commonly attributed to 'madness' or 'mental disorder.’ The idea of 'preservation' implies that something is at risk of being completely lost. As discussed in the previous section, it is common to both enchanted and disenchanted varieties of folk psychologies not to see method in the madness. In the former the person is 'possessed,' in the latter he is 'ill' due to a dysfunctional physical or psychological mechanism. Physical stance explanations of 'madness' are also present in societies where the institution of spirit possession is established. In this respect, the difference between such societies and disenchanted ones is that spirit possession preserves some intentionality where elsewhere the predominant option would be a physical explanation. Note that the issue here concerns the resources of an everyday folk psychology, and not of a theoretically driven account that may render behaviour understandable.

## Conclusion

Consideration of the connections between spirit possession, personhood, and intentionality afforded a novel perspective on spirit possession and a developed understanding of the intentional stance. Understanding spirit possession and intentionality in this light suggests the following insight: Centuries before the modern disciplines of psychoanalysis, phenomenological-psychopathology and the philosophy of mental health came on stage and tried to address the prejudices of folk psychology by restoring meaning to 'madness,' the social institution of spirit possession had been preserving the intentionality of socially inappropriate and un-understandable behaviour. By contra-posing a world of human-persons to that of spirit-persons and by allowing the latter the capacity to affect, or be the agent of, human behaviour, social deviance is not seen, at least initially, as 'mental disorder.’ The representation of spirits as agents with beliefs, desires and goals lends to socially problematic behaviour an intentionality that it may otherwise lack. And this allows, in some cases, for the explanation and prediction of behaviour. The exposition and analysis offered in this paper raise a question of importance with which I shall conclude: Is the spirit stance (and hence some intentionality) preferable to the physical stance (and therefore no intentionality) in terms of the social explanation of apparently meaningless behaviour in contexts where these are the predominant options? It is perhaps in understanding the issues relevant to thinking about this question, that some insight can be achieved into the value we place on meaning as such, and whether preserving meaning is a sufficient reason for us to relax our conceptions of agency and personhood.
